# The Role of CMV Infection in Primary Lesions, Development and Clinical Expression of Atherosclerosis

**DOI:** 10.3390/jcm11133832

**Published:** 2022-07-01

**Authors:** Carmen Valentina Cristescu, Sophie Alain, Simona Maria Ruță

**Affiliations:** 1 School of Advanced Studies of the Romanian Academy (SCOSAAR), Romanian Academy, 010071 Bucharest, Romania; 2 National Center for Cytomegalovirus Research, UMR 1092, 87042 Limoges, France; sophie.alain@unilim.fr; 3 Virology Department, “Carol Davila” University of Medicine and Pharmacy, 020021 Bucharest, Romania; simona.ruta@umfcd.ro; 4 Stefan S. Nicolau Institute of Virology, 030304 Bucharest, Romania

**Keywords:** cytomegalovirus, atherosclerosis, cardiovascular disease

## Abstract

The number of deaths related to cardiovascular disease is increasing every year, despite all available therapies and the aggressive campaigns for lifestyle modification and prevention of risk factors. Atherosclerosis is a complex process underlying cardiovascular disease. Cytomegalovirus (CMV) is often associated to atherosclerosis and its clinical expression such as coronary heart disease, stroke, or peripheral artery disease. CMV infection may promote acute atherosis within placentas from women with preeclampsia and it may also accelerate atherosclerosis in HIV-infected and organ-transplanted patients. This review focuses on the current scientific evidence for the role of CMV infection in the development of acute atherosis and atherosclerosis from placentation throughout life.

## 1. Introduction

Atherosclerosis is the pathophysiological phenomenon underlying myocardial infarction, stroke, and peripheral artery disease. It is a progressive inflammatory process that can occur in people of all ages [1,2,3], even in childhood [4]. Atherosclerosis is characterized by the accumulation of lipids and secondary fibrosis of the arterial wall, followed by calcification or plaque instability and eventually plaque rupture with clinical events. Both the innate and adaptive immune responses play an important role in the evolution of the disease [5]. The process of plaque formation may extend through one or more decades or can be very fast, evolving within a few months, depending on the type and onset of the immune response. The mechanism of atherosclerosis is still unclear [3,5], although inflammation plays a crucial role and specific lifestyle-related risk factors have a high impact (smoking, dyslipidemia, diabetes, obesity, level of physical activity, and stress). Various infectious agents have been investigated as potential triggers or cofactors for atherosclerosis [6]. Thus, Helicobacter pylori induces chronic systemic inflammation by molecular mimicry. Periodontal pathogens can cause bacteriemia, leading to direct plaque invasion. SARS-CoV-2 generates a cytokine storm and plaque thrombosis. Human herpes simplex virus produces a proinflammatory state with lipid metabolism impairment. Hepatitis C virus increases the expression of cardiac and inflammatory biomarkers. Streptococcus pneumoniae, Chlamydia pneumoniae, and Mycoplasma pneumoniae may induce persistent pro-inflammatory state and platelet activation with subsequent plaque instability [7]. Among the plethora of bacteria and viruses, CMV was frequently detected in both atherosclerotic plaques and in healthy arteries, and its persistence is related to development overtime of atherosclerosis [8].

CMV is an ubiquitous beta herpesvirus, with a global prevalence in general population estimated at 83% [9] and a regional prevalence correlated with the socio-economic level of each country. During the last decades, CMV was incriminated in plaque formation and cardiovascular disease [10] due to its capacity for infecting endothelial cells, macrophages, dendritic cells, fibroblasts, and even smooth muscle cells, and its lifetime latency in different cellular types including monocytes and hematopoietic stem cells, with multiple reactivations that can trigger a chronic inflammatory status [10,11,12,13].

## 2. Cytomegalovirus and Cardiovascular Disease

### 2.1. Evidence of CMV’s Role in Plaque Formation and Cardiovascular Disease

#### 2.1.1. Epidemiological Studies

The first observations of a possible correlation between CMV and atherosclerosis were made almost 30 years ago. The presence of anti-CMV IgG antibodies within the sera and the presence of CMV antigens within vascular smooth muscle cells were associated with different stages of plaque formation in patients undergoing vascular surgery at that time [14,15]. Since then, the ongoing research on the implications of CMV infection in cardiovascular disease generated rather variable results, suggesting the importance of the study design and population sampling (Table 1).

Most CMV serology-based studies in patients at risk for cardiovascular diseases have delivered controversial results, except for a certain population group such as patients with diabetes. High titers of anti-CMV IgG antibodies correlate to atherosclerosis in type 2 diabetes mellitus patients, after adjustment of other common cardiovascular risk factors [16]. Patients with diabetes mellitus have an increased risk and an accelerated rate of development of atherosclerosis [17]. There might be a bi-directional link between CMV infection and diabetes, as patients with diabetes mellitus have an impaired antiviral immune response [18] that might promote CMV reactivation, while chronic CMV infection is associated with glucose regulation [19] and new-onset type 2 diabetes mellitus [20]. Nevertheless, all these complex interactions need a better understanding and more research studies in order to clarify the link between CMV infection and diabetes and cardiovascular disease.

CMV direct detection by DNA or antigen identification strongly associates viral infection with atherosclerosis and cardiovascular disease as an end-point. Recent studies showed a higher viral load in STEMI patients compared to controls [21] and a direct relationship between T cells activation and the number of CMV-DNA copies inside the atherosclerotic plaques in patients with peripheral artery disease [22]. A higher number of CMV-DNA copies were detected in patients with preexistent cardiovascular risk factors and were associated to acute coronary syndrome, suggesting that CMV reactivation may lead to the progression of atherosclerotic lesions, for example, transforming stable angina into unstable angina or myocardial infarction [23]. CMV-DNA was found in aortic plaques, but not in normal artery samples obtained from candidates for coronary artery by-pass graft [24]. Immunohistochemical studies demonstrated the presence of CMV pp65 antigen [25] in plaques obtained from patients with atherosclerosis undergoing vascular surgery [26].

Differences in the worldwide seroprevalence of CMV, as well as in the sample type (blood, atheroma plaques, and vascular wall fragments) and detection technique (serology, immunofluorescence, PCR alone or combined) can account for the sometimes controversial data. Overall, direct detection of viral antigens and/or DNA is a better proof of CMV association with atherosclerosis or cardiovascular disease, compared to serology alone.

#### 2.1.2. HIV Associated CMV Infection and Atherosclerosis

Asymptomatic CMV replication can trigger immune system activation and perpetuate an inflammatory environment that favors the early development of atherosclerosis. This is more evident in immunosuppressed individuals, either transplant recipients or people living with HIV (PLWH). Previous studies in HIV-infected individuals have suggested a link between CMV reactivations and an increased intima-media thickness, demonstrated by higher levels of specific anti-CMV antibodies and CMV-specific T-cell responses [31,32]. Probably, the two pathogens act synergistically to promote the complex process of plaque formation [33]. Macrophages and monocytes involved in the initial “traditional” atherosclerotic lesions are reservoirs for both HIV and CMV and the activation of these cells, with foamy cells formation, represent the main mechanism in plaque initiation. Early stages of atherosclerosis have been detected by coronary computed tomography angiography (CCTA) in HIV individuals, who are at high risk of thrombosis and plaques rupture, leading to HIV-associated acute coronary syndrome [34]. Noncalcified, inflammatory plaques that can lead to myocardial infarction and stroke have been also detected in PLWH [35]. Cardiovascular disease remains an important cause of non-AIDS-related morbidity and mortality during HIV infection even under continuous cART treatment [36]. PLWH successfully treated with cART, with low or undetectable HIV replication, still express regulatory viral proteins (Tat and Nef) that may alter monocyte/macrophages cell function [35], possibly triggering subclinical episodes of CMV reactivation. These, in turn, will maintain or amplify the status of chronic inflammation, T cell activation, and immune dysregulation already present in HIV-infected patients, even in those under long-term suppressive cART or in elite controllers. Supporting this hypothesis, a small randomized placebo controlled trial on the effect of valgancyclovir, a potent antiviral drug, showed a significant decrease in both CMV DNA and the level of CD8 T cell activation in immunosupressed HIV-infected patients under supressive cART [37]. Moreover, chronic CMV infection is characterized by an unusual expansion of specific memory T cells and is a potent trigger for a particular phenotype of CD4 cells with cytotoxic activity that migrate toward the vascular endothelium, playing an important role in the initial vascular lesions and in the progression of atherosclerosis [38]. This process might be accelerated in HIV-infected patients who have a high level of circulating CD8 T cells with increased expression of CX3CR1, the receptor of vascular-endothelium homing chemokine that can be attracted to endothelial cells inducing persistent activation and dysfunction [39].

#### 2.1.3. Cytomegalovirus and Plaque Formation in Pregnancy

Atherosclerosis may start during placentation, increasing the lifelong risk of atherosclerotic disease in women developing preeclampsia, a hypertensive pregnancy disorder [40,41,42]. CMV is the leading cause of neonatal congenital infections worldwide and is strongly associated with neurological sequelae in newborns and possibly associated with arterial hypertension, a mechanical blood flow condition that damages endothelial cells leading to preatherosclerotic lesions [43]. Based on the above-mentioned observations, we searched for studies that investigated the possible role of CMV in preeclampsia. Several studies looked at the impact of CMV infection on pregnancy complications associated with hypertension, but the results are quite controversial. Interestingly, some studies suggest that the medium-term risk of cardiovascular events in women that develop a hypertensive pregnancy disorder and their offspings is double compared to controls [40,44]. A significant association was found between CMV IgG sero-positivity and innate immune response in early-onset preeclampsia and the presence of HELLP (H: hemolysis, EL: elevated liver enzymes, LP: low platelet count) syndrome [45]. On the contrary, a small case-control study on hypertensive pregnancies, as well as a meta-analysis that included 2734 women with preeclampsia and 3424 healthy controls concluded that there is no significant relation between CMV infection (evaluated by both PCR and serology) and the onset of preeclampsia [46]. Nevertheless, it is interesting that acute atherosis occurs more often in women with preeclampsia [47] compared to normal pregnancies and other pregnancy complications like growth restriction, spontaneous preterm labor, or fetal death.

The hypothesis of a possible impact of CMV in vascular remodeling and acute atherosis lesions with maternal and fetal long-term impact on cardiovascular disease is very interesting and opens a new research direction on non-conventional risk factors for cardiovascular disease in young and very young patients.

## 3. CMV Mechanisms of Action in Atherosclerosis

**Figure 1:**
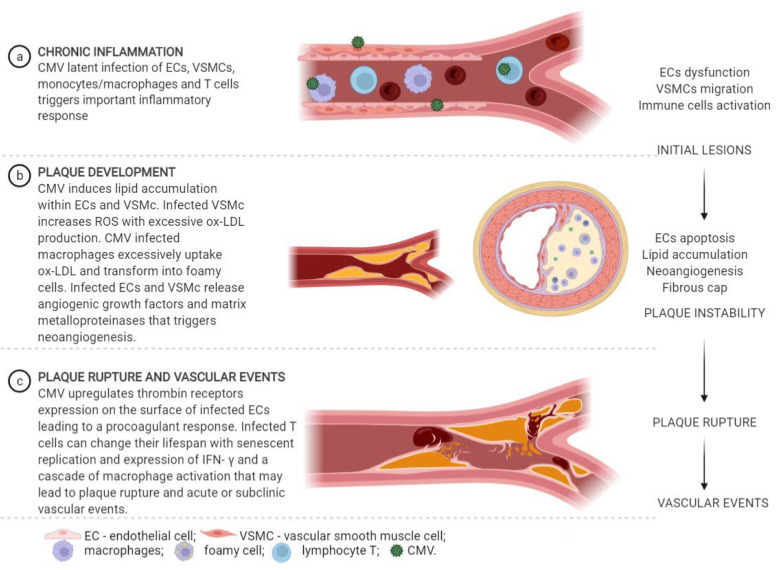
CMV mechanism in atherosclerosis (Created in BioRender.com).

Ever since it was used for the first time in 1755, the term atheroma describes a complex process involving both chronic inflammation and lipid accumulation, associated with vascular smooth muscle cells proliferation within the intimal layer of the blood vessel [48]. The potential mechanism by which CMV might promote atherogenesis remains poorly understood. However, CMV has a concerted impact on the main pathways involved in atherogenesis (Figure 1).

### 3.1. Oxidative Stress, Lipidogenesis and Endothelial Injury

Oxidized low-density lipoproteins (ox-LDL), which play a crucial role in the early events of atherogenesis, are preferentially recognized by scavenger receptors on macrophages and vascular cells. During CMV infection, the expression of type-B scavenger receptor on the surface of macrophages is upregulated [49]. Reactivation of the latent CMV infection in endothelial cells recruits macrophages and neutrophils by secreting chemoattractant and adhesion factors (MCP-1, VCAM-1, ICAM-1, and CXC), promoting internalization of oxLDL and foamy cells formation. CMV seems to upregulate several important pro-atherogenic molecules responsible for LDL and VLDL cellular uptake and cholesterol synthesis (NPC1L1, HMGCS1, HMGCR, LRP10, 11, 12, and SCARB) and downregulates anti-atherogenic proteins (ApoA1, ApoM, and ApoH) [50]. CMV-infected endothelial cells inhibit angiogenesis and promote abnormal vessel formation, a very important phenomenon already demonstrated in congenital CMV infection [51,52,53]. The expression of the receptor beta for PDGF (platelet-derived growth factor), an essential factor for the development and the plasticity of the vascular system, is upregulated by HCMV infection, stimulating atherogenesis [54]. CMV UL122 and US28-derived protein are homologous to an 11-aminoacid sequence of HSP60 that seems to produce endothelial cells apoptosis as an early event in atherogenesis [55]. The homology of pUS28 to HSP60 can also determine smooth muscle cells’ migration within vascular intima [55]. In vitro, CMV infects vascular smooth muscle cells, resulting in important alterations in the expression of lipid metabolism genes with intracellular cholesterol accumulation [56], possibly by downregulation of SSBP1, which is an important protein involved in wound repair [57].

### 3.2. Vascular Remodeling

A series of CMV-encoded proteins stimulate inflammation and vascular remodeling (Table 2).

Antibodies against CMV UL94, a region that encodes the viral tegument protein, are involved in the modulation of thousands of endothelial cells transcripts like adhesion molecules, growth factors, colony-stimulating factors, chemokines involved in leukocytes attraction, angiogenesis, and fibrosis [68]. CMV protein UL7 presents high structural and functional homology to CEACAM1, suggesting a direct involvement of CMV in vasculogenesis [58]. A stable domain in the structure of HCMV UL7 gene is responsible for STAT3/ERK1 MAP signal pathway activation. CMV can bind platelets using TLR2, generating the synthesis of proinflammatory CD40L, IL 1B, and proangiogenic VEGF, and further activating platelets, a cascade that can precipitate atherogenesis [69]. A recent in-vitro study published in 2020 suggests that chronic CMV infection downregulates endothelin 1 (ET-1) expression in endothelial and smooth muscle cells, thus providing additional evidence for the role of this pathogen in vascular impairment [70].

CMV itself can express viral cytokines and chemokines that might play a pivotal role in the angiogenesis and promotion of an inflammatory environment. An extensive homology between CMV and endothelial cell proteins were described and many immune cross-reactions caused by this molecular mimicry were incriminated in endothelial cells apoptosis, in the up-regulated expression of different cytokines and in the pathogeny of vascular damage in autoimmune diseases [68,71].

### 3.3. miRNA Regulation

During the last decade, small, highly-conserved, noncoding single-stranded RNA fragments called micro RNA (miR) were described as promoters and potential biomarkers of atherosclerotic lesions [72]. miRs were widely investigated and determined to be regulating different cell functions in atherosclerosis. They play a crucial role in all stages of atherosclerotic process, from the initial endothelial injury and lipid accumulation to angiogenesis, calcification, and thrombosis [72,73]. miR-21, for example, increases NOS phosphorylation and inhibits PPARa and Bcl2, leading to apoptosis and endothelial cells injury [73]. Elevated levels of miR-338-3p were identified in patients with atherosclerosis and were linked to ox-LD-induced apoptosis within endothelial cells [50]. Likewise, miR-126 and miR-127 are related to cell apoptosis by increasing vascular cell adhesion protein1 (VCAM1) and NAD-dependent deacetylase sirtuin-1 [73]. An impressive number of CMV-encoded miR were also identified [74] and it was postulated that they can also participate in vascular remodeling and endothelial injury [50]. A summary of some pro-atherosclerotic CMV-encoded miRs according to cell type function regulation is described in Table 3.

### 3.4. Animal Model Studies

Due to its species specificity, human CMV in-vivo studies are quite limited and animal models are frequently used to mimic genes/proteins and the secretome of human CMV. Of all animal models, rat and murine models have the highest homology to human CMV [81,82]. Rat and murine infection models produce acute as well as latent infection, with an important symptomatology in immunosuppressed animals. In murine models of atherosclerosis, CMV promotes a rapid course of transplant vascular sclerosis and induces upregulation of a series of genes involved in both angiogenesis and wound repair [83] and promotes an increased cytokine expression in the infected aortic samples [84]. CMV viral transcripts can modulate the host immune response and apoptosis [71], promoting endothelial damage and thus, atherosclerosis. For example, UL122 expressed on endothelial cells during early stages of infection show homology to connexin 45 and integrin alpha 3 and 6, while US28 also expressed on endothelial cells surface shows homology to integrin alpha 6 (CD49f) and seems to be involved in CMV reactivation from latency [68]. CMV IE2 gene delays smooth muscle cells apoptosis by upregulating the expression of antiapoptotic proteins Mcl-1 and Bcl-2 and was linked to a myocardin-induced transcriptional program responsible for aortic smooth muscle cells proliferation in rats’ aortas [85]. Furthermore, the role of CMV in cardiovascular disease seems to extend from a possible risk factor for atherosclerosis to direct myocardial pathology, as a recent animal model study demonstrates direct murine CMV infection of myocardial cells, with tachycardia and hypertrophy, suggesting CMV reactivation within the heart and vasculature [86]. The same murine model study showed viral DNA persistence within myocardial cells up to 100 days post-acute infection, but with a viral gpB gene expression of only 35 days post-infection, suggesting that CMV latency may start within the heart after acute infection [86]. Murine CMV has been associated with myocardial fibrosis, synus tachicardia and ventricular hypertrophy in chronically infected mice [87]. The results of these animal studies are additional proof for a possible role of CMV infection in atherosclerosis, cardiovascular disease, and direct myocardial injury. 

## 4. Conclusions

According to the World Health Organization, cardiovascular disease is the leading cause of death worldwide. Despite all efforts in preventing the conventional risk factors, the number of deaths related to myocardial infarction, stroke, peripheral artery disease, or hypertensive pregnancy disorders is increasing. The involvement of infectious agents in the mechanisms of atherosclerosis has been studied for more than two decades. The association between CMV, atherosclerosis, and cardiovascular diseases is still controversial and needs to be clarified. Due to its ability to infect almost all cell types and its secretome largely based on proinflammatory cytokines, chemoattractant, and vascular growth factors, CMV triggers plaque formation by a complex mechanism of endothelial injury, alteration of lipid metabolism with lipid deposition, vascular smooth muscle cells proliferation and migration, as well as disruption of coagulation mechanism and thrombosis.

This review highlights that CMV infection may be associated with acute atherosis, atherosclerosis, and/or cardiovascular disease in almost all population groups. It may produce vascular injuries eversince placentation and then continue to maintain a chronic inflammation status with subsequent vascular impairment and cardiovascular events later in life, in both healthy and immunosuppressed patients. Direct detection of CMV’s DNA or antigens in atherosclerotic patients is the best proof for a possible role of this ubiquitous virus in plaque development. In order to answer if there is a role as an independet risk factor for CMV in atherosclerosis and cardiovascular disease, complex multicentric cohort studies enrolling newborns, children, adolescents, young adults, adults, and elderly should be considered. This would allow a better understanding of the role played by chronic CMV infection and reactivation in atherogenesis and cardiac disease, in a very exciting era, in which new therapeutic and prophylactic strategies including vaccination are already in progress for this infectious agent.

## Figures and Tables

**Table 1:** Associations between CMV, atherosclerosis, and cardiovascular disease.

Authors	Reference	Population	CMV Determinant	Outcome	Association	Study Type
Adam et al.	[14]	157 caucasian male undergoing vascular surgery for atherosclerosis	Serology	PAD	YES	Case-control
Melnick et al.	[15]	132 arterial tissue samples from atheroslerotic plaques of caucasian patients undergoing vascular surgery for atherosclerosis	IF pp65	PAD	YES	Prospective cohort
Jia et al.	[27]	3328 multiethnic patients but predominantly asians with CAD and PAD with or without surgical indication	Serology CMV DNA IF pp65	CAD and PAD	YES	Meta-analysis (control-case or nested control-case)
Nikitskaya et al.	[22]	71 ACS, 26 SCAD, 22 atheroslerotic plaques (PAD)	CMV DNA	CAD and PAD	YES	Case-control
Zhang et al.	[16]	222 hospitalized patient with type 2 diabetes mellitus	Serology CMV DNA	T2DM and ultrasound confirmed atherosclerosis	YES for latent infection only	Prospective cohort
Courivaud et al.	[28]	570 renal transplant recipients	Serology CMV DNA	CAD, PAD, stroke	YES	Prospective cohort
Betjes et al.	[29]	408 ESRD patients	Serology	CAD and PAD	YES	Retrospective cohort
Lebedeva et al.	[21]	33 STEMI patients	CMV DNA	IHD (STEMI)	YES	Case-control
Nikitskaya et al.	[23]	97 CAD patiens	CMV DNA	CAD	YES	Case-control
Heybar et al.	[24]	55 CABG—normal and atherosclerotic samples	CMV DNA	CAD—CABG	YES	Case-control
Wang et al.	[25]	15 paraffin-embedded peripheral artery specimens from patients with ATS	IHC	PAD	YES	Prospective cohort
Ibrahim et al.	[26]	48 biopsies from atherosclerotic plaques	CMV DNA	PAD	YES	Case-control
Hamilton et al.	[30]	8531 white ethnic background with no prevalent CVD	Multiplex serology panel	CVD, IHD, stroke	NO	Prospective cohort

CAD—coronary artery disease, CABG—coronary artery by-pass graft, CVD—cardiovascular disease, ESRD—end-stage renal disease, IHD—ischemic heart disease, IHC—immunohistochemistry, PAD—peripheral artery disease, STEMI—ST-elevated myocardial infarction.

**Table 2:** CMV encoded proteins and the expression of humoral factors involved in atherosclerosis.

HCMV Protein	Function	Mechanism in Atheromatous Process
UL 7	Early-late gene that is cleaved to release glycosilated ectodomain [58]	Stimulates inflammation in endothelial cells by IL-6 expression and STAT and MAPK pathway activation [58] Increased similarity to the first Ig-like domain of the CEACAM protein family playing a key role in vasculogenesis [58,59]
UL 76	Unclear—highly conserved protein that may cleave nuclear proteins and modulate viral activation or repression [60,61]	Induces IL-8 expression by DNA damage [60]
UL 111A	Encodes human homologous IL-10 cmvIL-10 [62]	Activates CXCL12/CXCR4 and STAT3 signal pathways in monocytes, epithelial cells and fibroblasts [62]
UL 122	IE2 regulatory protein- modulates viral activation and reactivation from latency [55] A protein isoform IE86 encoded by UL122—DNA binding protein that regulates viral gene expression and recruits chromatin modeling enzymes. Essential for viral replication by transactivation of vital viral early promoters probably by direct DNA binding [63]	Homology to heat shock protein 60 (HSP60) and increased monocytes adhesion [55] Supresses the expression of proinflammatory citokines IFN β, RANTES, MIG, MCP2, IE1, GAPDH [64] and may play a protective role in atherosclerosis development
US 28	Expressed on cell surface with high homology to CC chemokine receptor CCR1 facilitating cell-cell fusion [65]	Homology to heat shock protein 60 (HSP60), determines smooth muscle cells migration [55]
UL 128	Envelope protein with immunoregulatory properties, responsible for monocytes and epithelial cells infection [66], thus virus replication and dissemination	Recruits monocytes/macrophages cells by chemoattraction and determines high expression of TNF-α and IL-6 [67]

**Table 3:** CMV encoded miR according to cell type function in atherogenesis.

CMV Encoded miR	Cell Type	Function	References
miR-217	Endothelial	Angiogenesis	[50,75]
miR-US25-1	Endothelial	Ox-LDL induced apoptosis	[76]
miR-UL112	Endothelial	Endothelial dysfunction by modulation of multiple signal pathways	[77,78]
miR-US4-5p	Macrophages	Apoptosis	[50,79]
miR-US33	Vascular smooth muscle cells	Apoptosis	[80]

## Data Availability

Not applicable.
